# Supplementary material for: CO2/CH4 Separation in Amino Acid Ionic Liquids, Polymerized Ionic Liquids, and Mixed Matrix Membranes
Source: Molecules. 2024 Mar 19;29(6):1357. doi: 10.3390/molecules29061357 (PMC10974021; doi:10.3390/molecules29061357)
Supplement: Supplementary file 1 [file molecules-29-01357-s001.zip › molecules-2871646-supplementary.pdf]

Amino Acid Ionic Liquids-Polymerized Ionic Liquids - Mixed Matrix Membranes for CO_2/CH_4 Separation

Gowri Selvaraj[1] and Cecilia Devi Wilfred[1,2,*]

1 Centre of Research in Ionic Liquids (CORIL), Institute of Contaminant Management (ICM), Universiti Teknologi PETRONAS, 32610 Sri Iskandar, Perak, Malaysia; selgowri@gmail.com

2 Fundamental and Applied Sciences, Universiti Teknologi PETRONAS, Seri Iskandar, 32610 Perak, Malaysia

* Correspondence: cecili@utp.edu.my

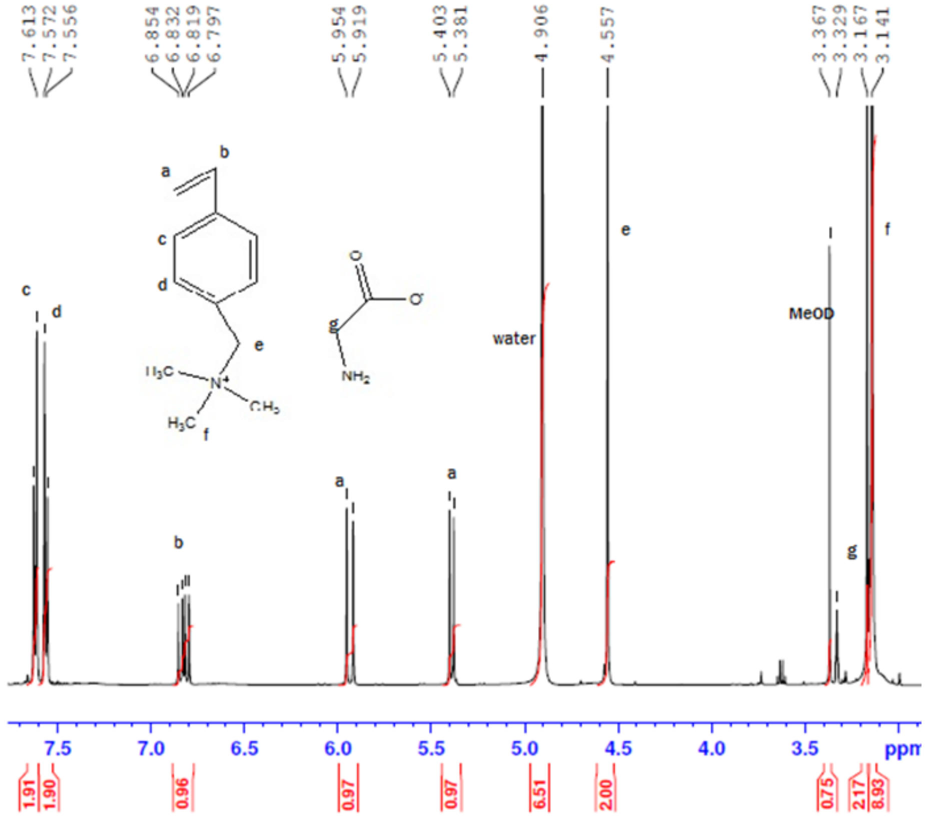

Figure S1: ¹H spectrum of [VBTMA][Gly].

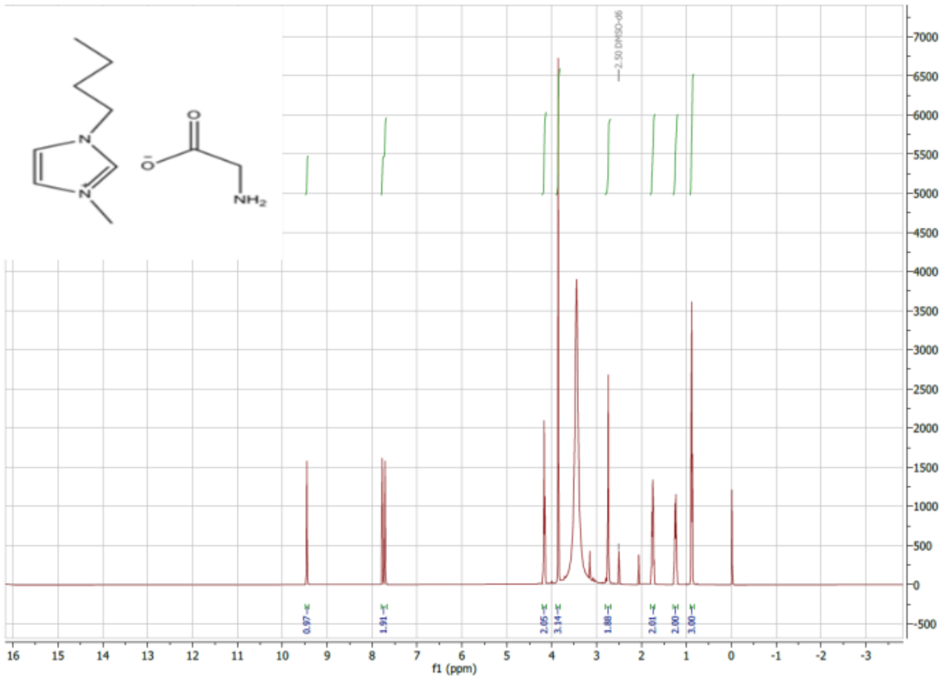

Figure S2: ^{1}H spectrum of [BMIM[Gly].
